# Supplementary material for: Introducing risk inequality metrics in tuberculosis policy development
Source: Nat Commun. 2019 Jun 6;10:2480. doi: 10.1038/s41467-019-10447-y (PMC6554307; doi:10.1038/s41467-019-10447-y)
Supplement: Supplementary file 1 — Supplementary Information [file 41467_2019_10447_MOESM1_ESM.pdf]

Introducing risk inequality metrics in tuberculosis policy development

Supplementary Information
Figures 1-12
Tables 1-11

Gomes *et al.*

Correspondence and requests for materials should be addresses to M.G.M.G. (email: gabriela.gomes@lstmed.ac.uk).

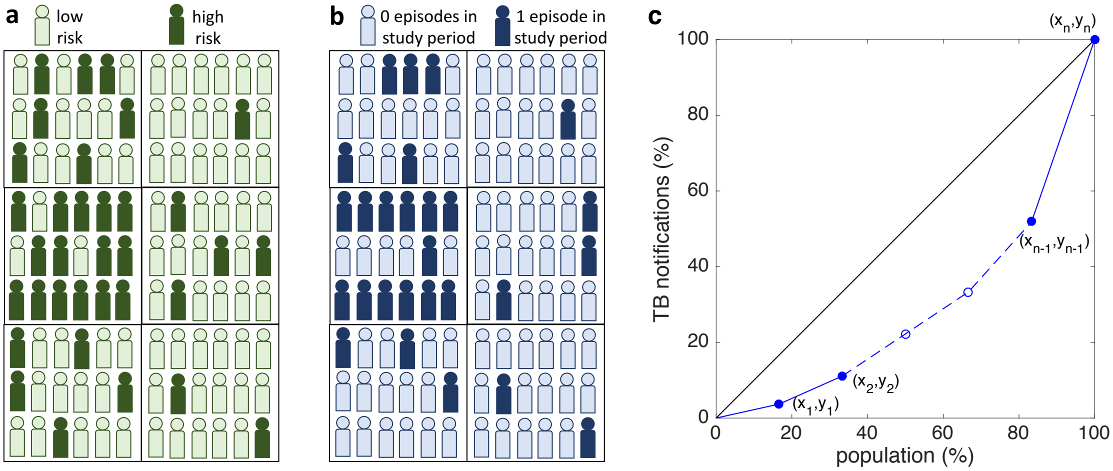

Supplementary Fig. 1: Lorenz curve from a nonuniform distribution of low and high risk individuals. A nonuniform distribution of low and high risk individuals (**a**) leads to variation in disease incidence across population divisions (**b**). In reverse, summary measures of risk inequality can be obtained from conveniently stratified incidence data: **c** depicts the construction of a Lorenz-like curve from incidence data in a hypothetical country partitioned into geographical divisions. Population divisions are ranked from lower to higher incidence and cumulative measures of population (x_i) and disease notifications (y_i) are calculated for every index i between 1 and the total number of divisions n. Lorenz curves are widely used in economics to summarize measures of inequality in the distribution of wealth and can be used in creative ways to describe distributions of population health.

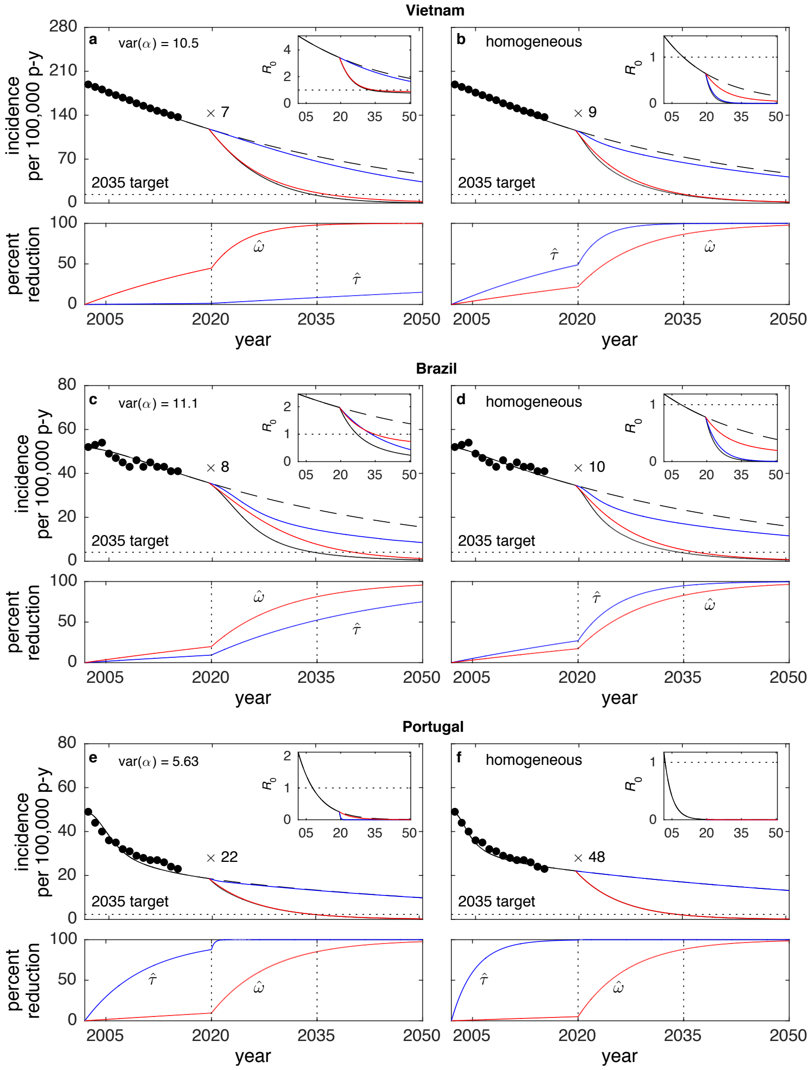

Supplementary Fig. 2: Model trajectories with heterogeneity in contact rates and gradual increases in successful treatment (τ) and declines in reactivation (ω). TB incidence from 2002 to 2015 (black dots) and model solutions under heterogeneous contact rates (**a**, **c, e**); homogeneous approximation (**b**, **d, f**). Initial parameter values calculated by adjusting the mean effective contact rates (β) to fit 2002 incidence rates: $\beta = 3.23\ \text{yr}^{-1}$ (**a**) or $\beta = 10.7\ \text{yr}^{-1}$ (**b**) in Vietnam; $\beta = 2.94\ \text{yr}^{-1}$ (**c**) and $\beta = 17.3\ \text{yr}^{-1}$ (**d**) in Brazil; $\beta = 4.66\ \text{yr}^{-1}$ (**e**) and $\beta = 17.1\ \text{yr}^{-1}$ (**f**) in Portugal. Incidence declines towards 2015 attributed to increasing successful treatment (τ) and reducing reactivation (ω): $\tau(t) = 2e^{r_\tau(t-2002)}$ and $\omega(t) = \omega_0 e^{r_\omega(t-2002)}$ (where $\omega_0 = 0.0039$ in Vietnam and $\omega_0 = 0.0013$ in Brazil and Portugal), with constant rates r_τ and r_ω estimated using MCMC (Supplementary Table 5). From 2020 onwards, the trajectories split to represent four scenarios: rates of parameter change are maintained (dashed black); scale r_τ and r_ω by a factor κ (represented as "$\times\ \kappa$") to meet WHO incidence target for 2035 (solid black); apply the same scale up efforts to r_τ only (blue) or r_ω only (red). The bottom plots in each panel represent the cumulative improvements in successful treatment and reactivation required to meet the targets calculated as $\hat{\tau}(t) = 1 - \tau(2002)/\tau(t)$ and $\hat{\omega}(t) = 1 - \omega(t)/\omega(2002)$, respectively. Clearance of infection upon successful treatment: $\theta = 1$. Other parameters as in Table 1. Model described by equations (1)-(5), and R_0 given by (6).

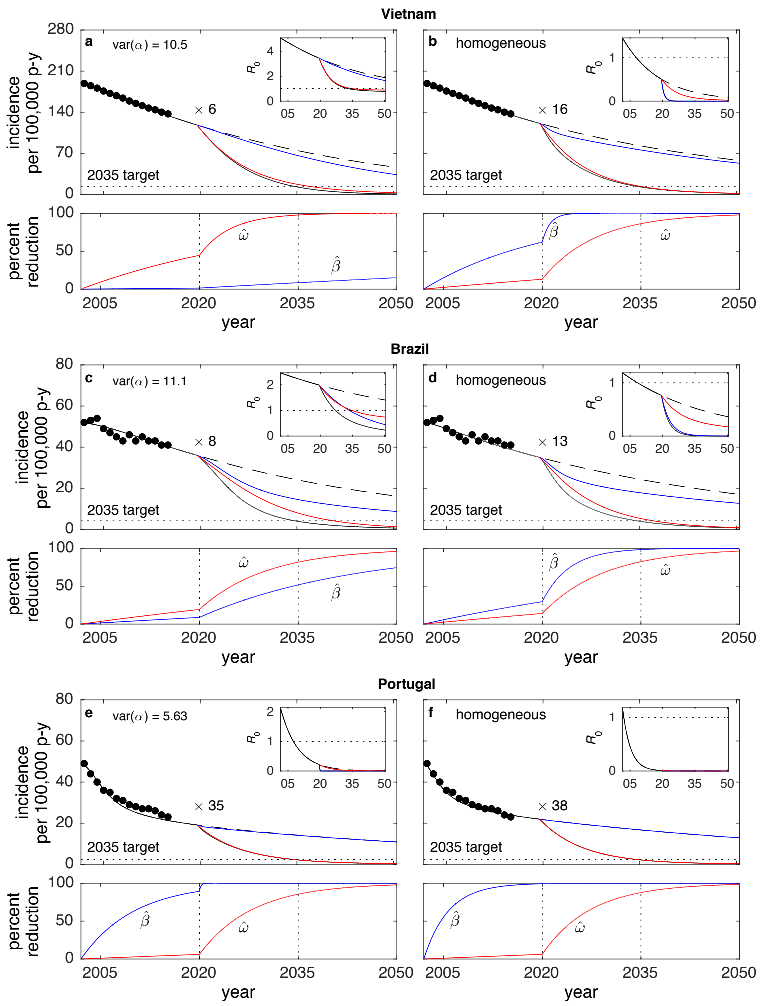

Supplementary Fig. 3: Model trajectories with heterogeneity in contact rates and gradual declines in effective contacts ($\boldsymbol{\beta}$) and reactivation ($\boldsymbol{\omega}$). TB incidence from 2002 to 2015 (black dots) and model solutions under heterogeneous contact rates (**a**, **c, e**); homogeneous approximation (**b**, **d, f**). Initial parameter values calculated by adjusting the mean effective contact rates (β) to fit 2002 incidence rates: $\beta_0 = 3.23\ \mathrm{yr}^{-1}$ (**a**) and $\beta_0 = 10.7\ \mathrm{yr}^{-1}$ (**b**) in Vietnam; $\beta_0 = 2.94\ \mathrm{yr}^{-1}$ (**c**) and $\beta_0 = 17.3\ \mathrm{yr}^{-1}$ (**d**) in Brazil; $\beta_0 = 4.66\ \mathrm{yr}^{-1}$ (**e**) and $\beta_0 = 17.1\ \mathrm{yr}^{-1}$ (**f**) in Portugal. Incidence declines towards 2015 attributed to reducing effective contacts (β) and reactivation (ω): $\beta(t) = \beta_0 \mathrm{e}^{r_\beta (t-2002)}$ and $\omega(t) = \omega_0 \mathrm{e}^{r_\omega (t-2002)}$ (where $\omega_0 = 0.0039$ in Vietnam and $\omega_0 = 0.0013$ in Brazil and Portugal), with constant rates r_β and r_ω estimated using MCMC (Supplementary Table 6). From 2020 onwards, the trajectories split to represent four scenarios: rates of parameter change are maintained (dashed black); scale r_β and r_ω by a factor κ (represented as "$\times\ \kappa$") to meet WHO incidence target for 2035 (solid black); apply the same scale up efforts to r_β only (blue) or r_ω only (red). The bottom plots in each panel represent the cumulative reductions in effective contacts and reactivation required to meet the targets calculated as $\hat{\beta}(t) = 1 - \beta(t)/\beta(2002)$ and $\hat{\omega}(t) = 1 - \omega(t)/\omega(2002)$, respectively. Clearance of infection upon successful treatment: $\theta = 1$. Other parameters as in Table 1. Model described by equations (1)-(5) and R_0 given by (6).

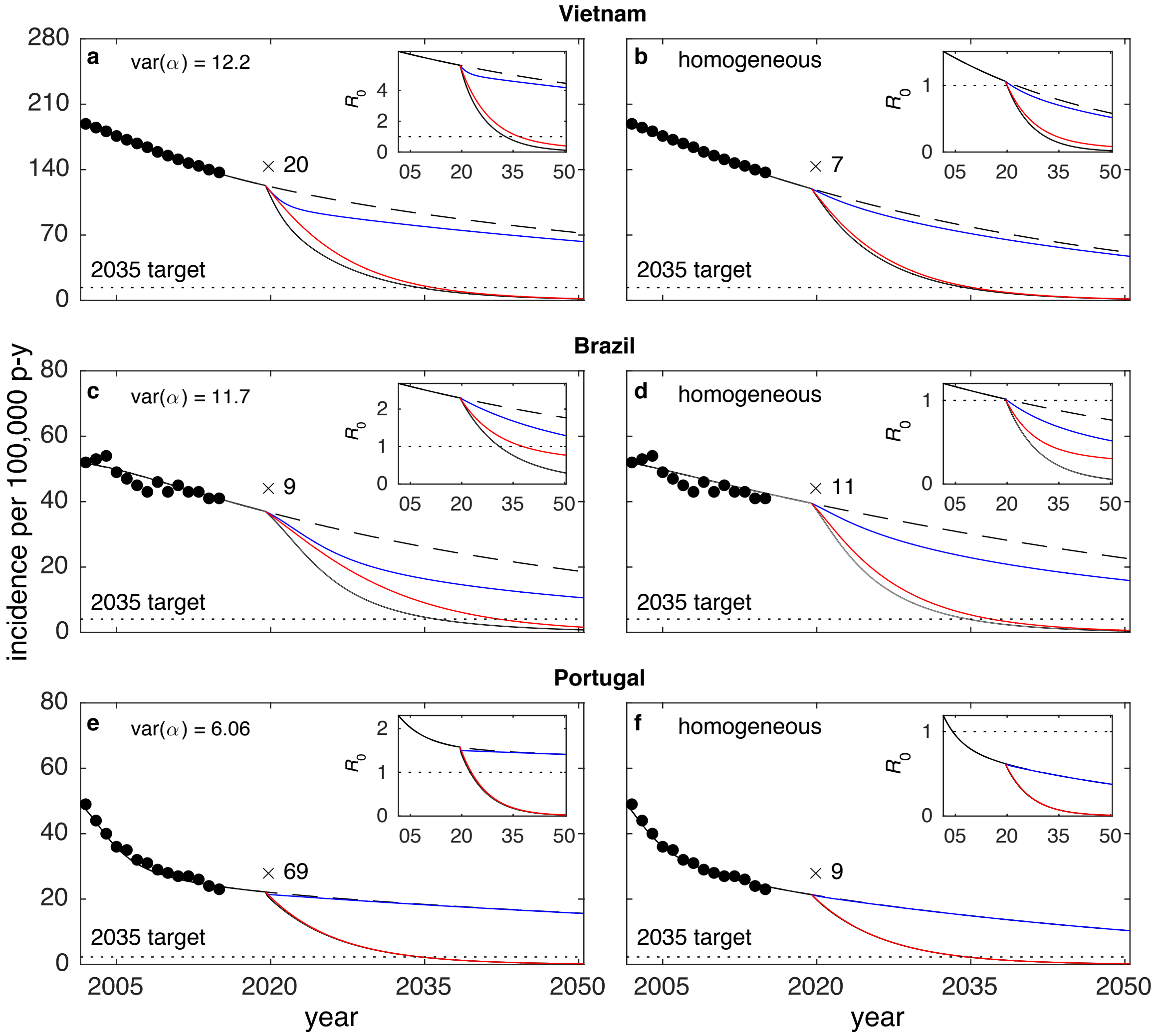

Supplementary Fig. 4: Model trajectories with heterogeneity in contact rates, gradual declines in disease progression (ϕ) and reactivation (ω), and no clearance of infection upon successful treatment ($\theta = 0$). TB incidence from 2002 to 2015 (black dots) and model solutions under heterogeneous contact rates (**a**, **c**, **e**); homogeneous approximation (**b**, **d**, **f**). Initial parameter values calculated by adjusting the mean effective contact rates (β) to fit 2002 incidence rates: $\beta = 2.78\ \mathrm{yr}^{-1}$ (**a**) or $\beta = 8.47\ \mathrm{yr}^{-1}$ (**b**) in Vietnam; $\beta = 2.78\ \mathrm{yr}^{-1}$ (**c**) or $\beta = 15.8\ \mathrm{yr}^{-1}$ (**d**) in Brazil; $\beta = 4.29\ \mathrm{yr}^{-1}$ (**e**) or $\beta = 15.7\ \mathrm{yr}^{-1}$ (**f**) in Portugal. Incidence declines towards 2015 (solid black curves) attributed to reducing disease progression (ϕ) and reactivation (ω): $\phi(t) = 0.05\mathrm{e}^{r_\phi(t-2002)}$ and $\omega(t) = \omega_0\mathrm{e}^{r_\omega(t-2002)}$ (where $\omega_0 = 0.0039$ in Vietnam and $\omega_0 = 0.0013$ in Brazil and Portugal), with constant rates r_ϕ and r_ω estimated using MCMC (Supplementary Table 8). From 2020 onwards, the trajectories split to represent four scenarios: rates of parameter change are maintained (dashed black); scale r_ϕ and r_ω by a factor κ (represented as "$\times\ \kappa$") to meet WHO incidence target for 2035 (solid black); apply the same scale up efforts to r_ϕ only (blue) and r_ω only (red). Other parameters as in Table 1.

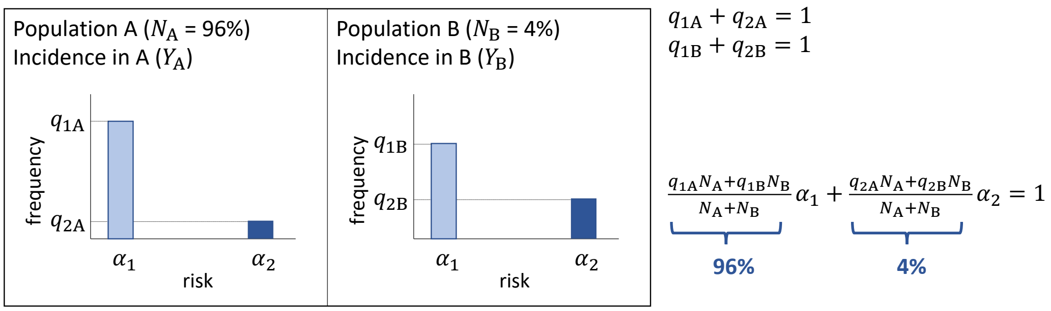

Supplementary Fig. 5: Metapopulation diagram. A metapopulation consisting of two populations (A and B), each characterized by an individual risk distribution. Populations (or patches) in this model are composed of individuals drawn from a common pool of individuals with high (α_2) and low (α_1) risk (in proportions 4% and 96%, respectively), and what characterizes each patch is the fraction of its individuals who are high risk. Assuming a single effective contact rate (β) for the entire metapopulation, we vary the proportion of individuals in A who are high risk (q_{2A}) and calculate the corresponding proportion in B (q_{2B}). This describes a family of metapopulation models, parameterized by the proportion of high-risk individuals in one of the patches, that can be completely resolved to match the incidence and RIC for each study country.

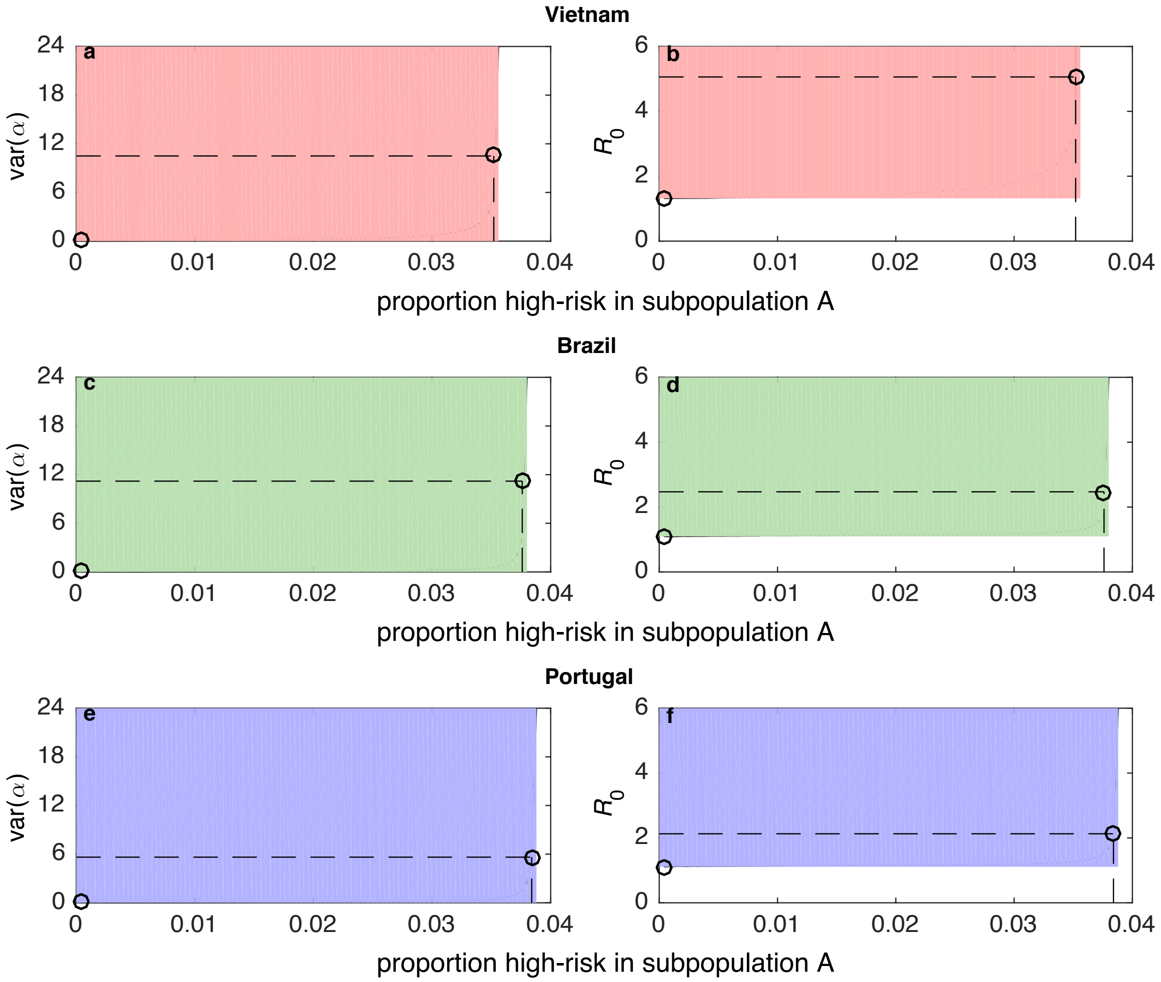

Supplementary Fig. 6: One-parameter family of metapopulation models with heterogeneous contact rates. Each point along a solid curve represents one model that produces country incidences in agreement with RIC values in Fig. 1. Dashed lines and the corresponding circle highlight a model with variance in individual risk as in this study. The other circle marks a more common metapopulation model where individuals differ between but not within patches. Metapopulation diagram in Supplementary Figure 5.

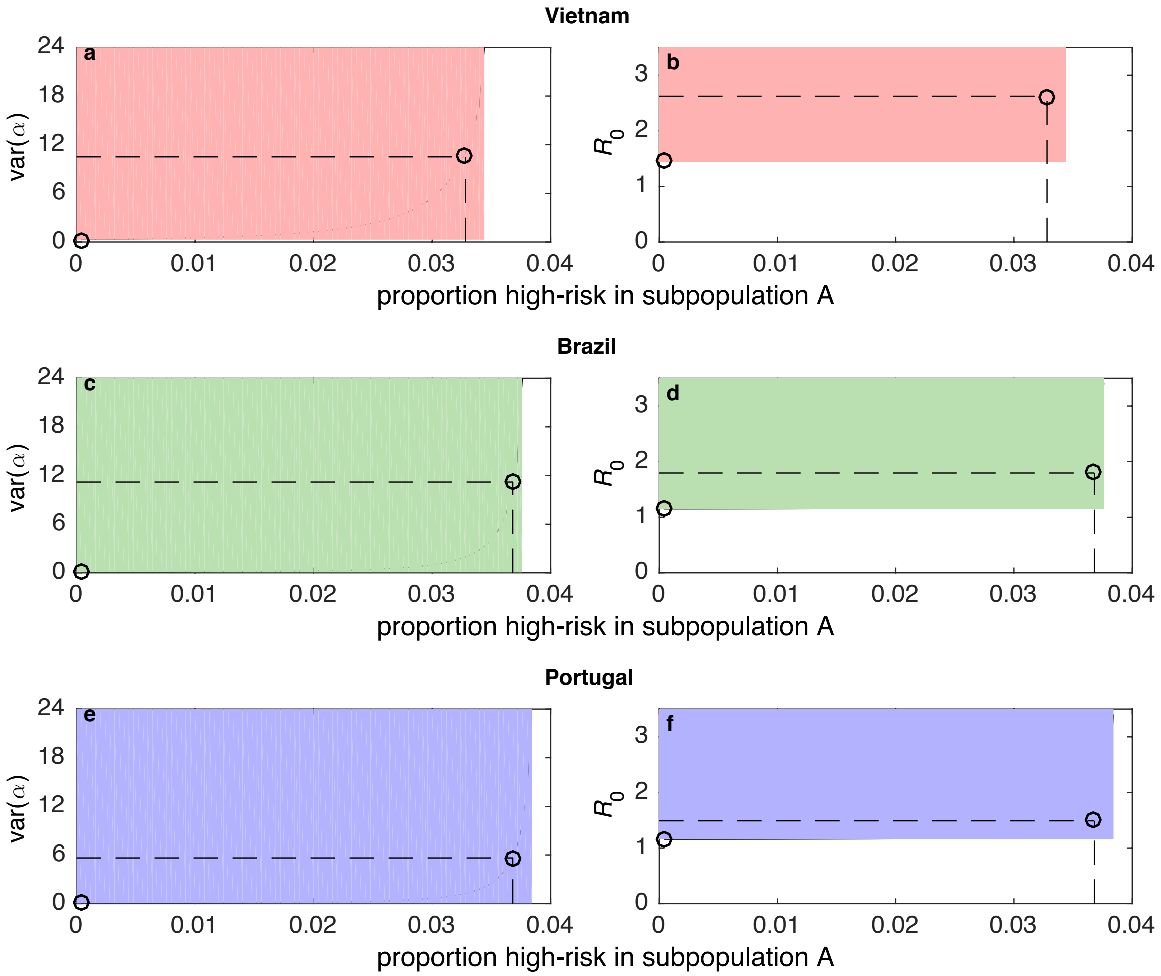

Supplementary Fig. 7: One-parameter family of metapopulation models with heterogeneous susceptibility to infection. Each point along a solid curve represents one model that produces country incidences in agreement with RIC values in Fig. 1. Dashed lines and the corresponding circle highlight a model with variance in individual susceptibility as in this study. The other circle marks a more common metapopulation model where individuals differ between but not within patches. Metapopulation diagram in Supplementary Figure 5.

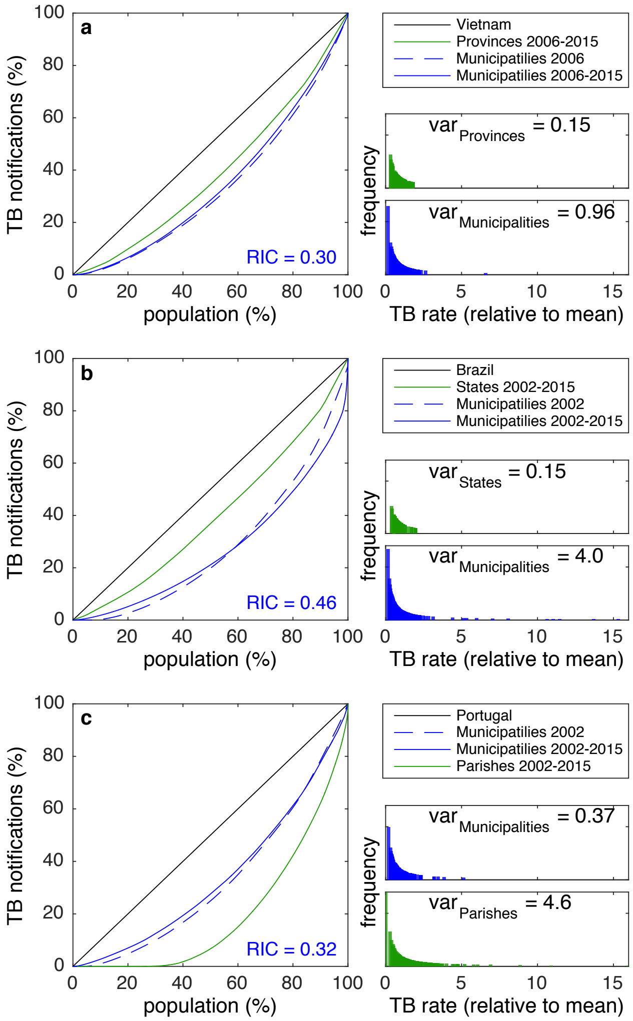

Supplementary Fig. 8: Risk inequality metrics under alternative administrative divisions. Lorenz curves constructed from notification data covering various time periods and administrative divisions. Level 2 divisions (**a**, 697 municipalities in Vietnam; **b**, 5127 in Brazil; **c**, 308 in Portugal) are shown in blue (solid using the number of years available to this study [2006-2015 in Vietnam, and 2002-2015 in Brazil and Portugal], and dashed using only the first year in each series). Green curves correspond to alternative administrative levels (64 provinces in Vietnam [level 1]; 27 states in Brazil [level 1]; 3281 parishes in Portugal [level 3]). Notification rate distributions corresponding to blue and green solid curves are plotted together with the respective variances. Two-risk group discretizations were used in the paper while here we show 100-risk group discretizations of the same distributions. The entire analysis could be conducted with more finely resolved distributions if desired by simply increasing the dimension of the dynamical system accordingly.

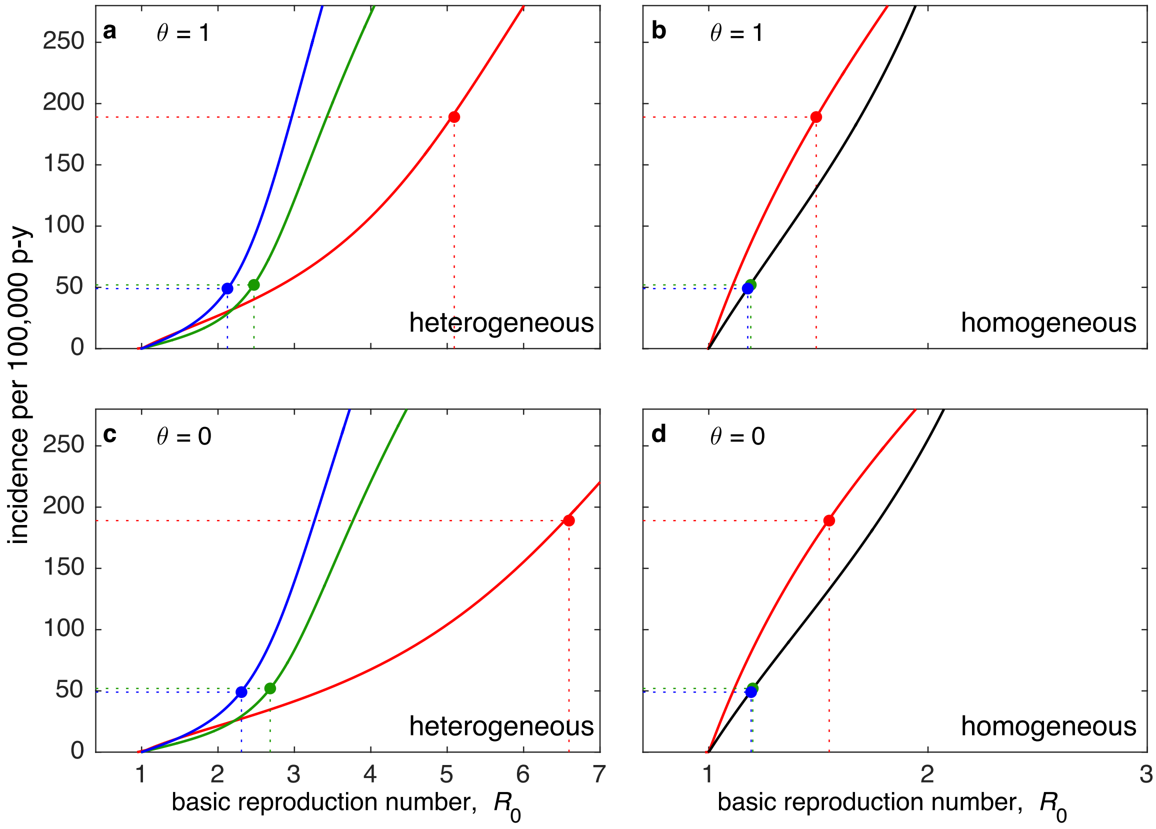

Supplementary Fig. 9: Endemic equilibria with heterogeneity in contact rates parameterized by the effective contact rate ($\boldsymbol{\beta}$) and plotted against $\boldsymbol{R_0}$. Equilibrium solutions of the heterogeneous model (1)-(5) with bacterial clearance upon successful treatment ($\theta = 1$) and risk distributions displayed in Fig. 2 for Vietnam (red), Brazil (green), and Portugal (blue) (**a**); respective mean field approximations (**b**). **c**, **d,** Same procedure with no bacterial clearance upon successful treatment ($\theta = 0$). Dots mark incidence rates in 2002 as reported by WHO and their positioning on the curves gives the initial value of β for each country. R_0 is given by (6), and other parameters are as in Table 1.

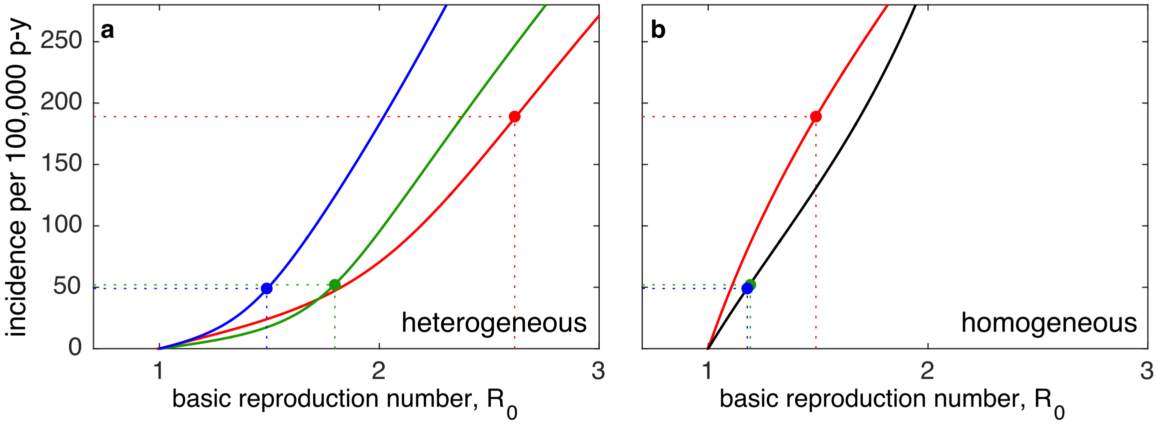

Supplementary Fig. 10: Endemic equilibria with heterogeneity in susceptibility to infection parameterized by the effective contact rate (β) and plotted against R_0. Equilibrium solutions of the heterogeneous model (1)-(4) and (7) with bacterial clearance upon successful treatment ($\theta = 1$) and risk variances provided in Fig. 6 for Vietnam (red), Brazil (green), and Portugal (blue) (**a**); respective mean field approximations (**b**). Dots mark incidence rates in 2002 as reported by WHO and their positioning on the curves gives the initial value of β for each country. R_0 is given by (8), and other parameters are as in Table 1.

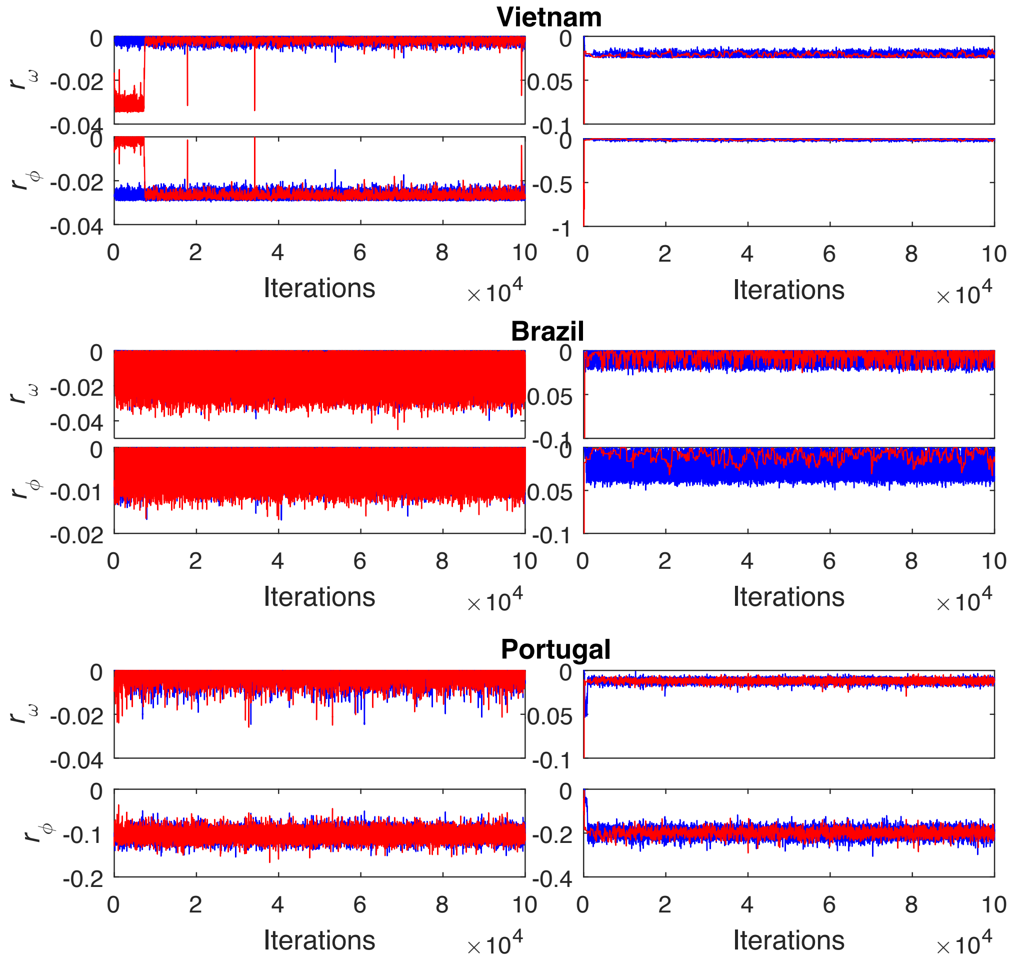

Supplementary Fig. 11: MCMC posterior parameter values. Comparing posterior parameter distributions for two independent chains with different starting values (blue: as described in main text; red: an over-dispersed starting value) for heterogeneous contact rates (left) and homogeneous (right) models in Fig. 4. Both chains traverse the parameter space in the same way which indicates convergence.

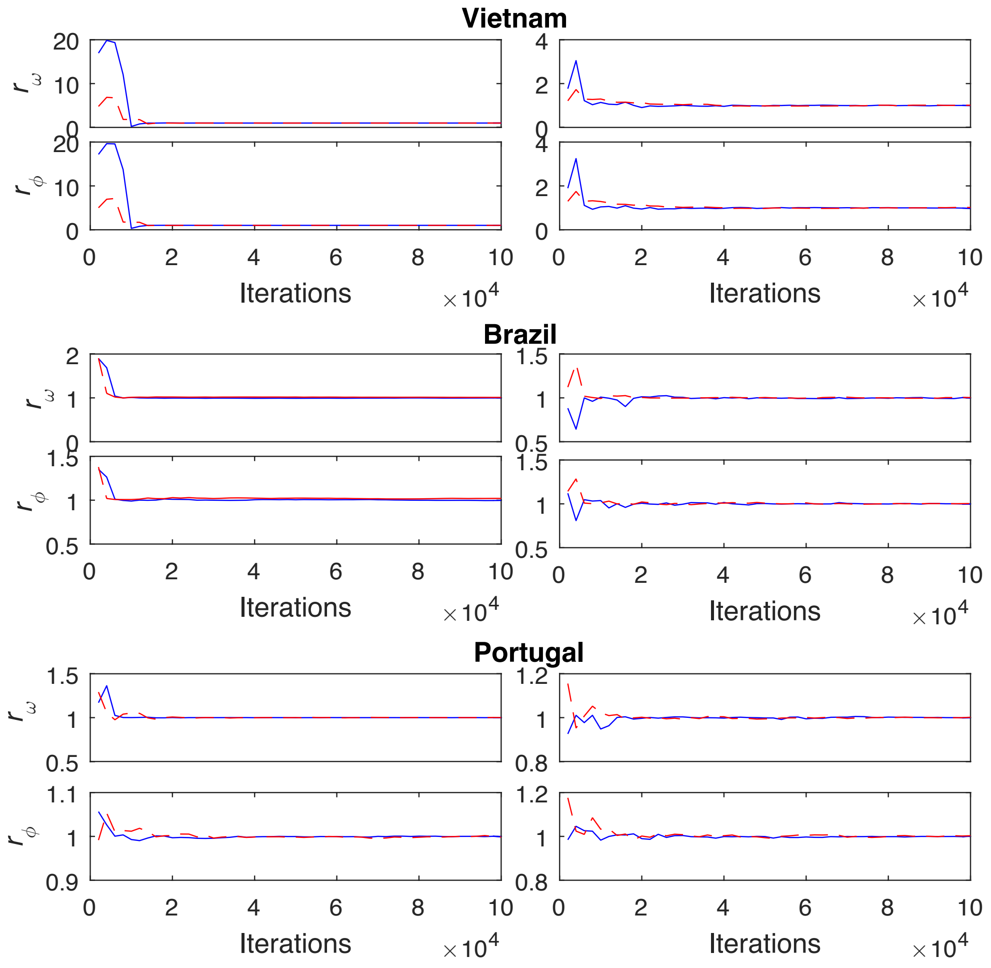

Supplementary Fig. 12: MCMC Gelman-Rubins-Brooks potential scale reduction factor. The Gelman-Rubins-Brooks potential scale reduction factor (psrf) for the posterior distributions of two independent chains with different starting values (blue: as described in main text; red: an over-dispersed starting value) for heterogeneous contact rates (left) and homogeneous (right) models in Fig. 4. As the number of iterations increase the psrf of the chains decline to values close to 1 which suggest that both chains are no longer influenced by their starting values. We consider a burn-in of 2×10^4 iterations in all the runs as the chains seems to stabilize after this.

Supplementary Table 1: Moving targets. Values of incidence and r_ω (decay rate in reactivation) used in Fig. 3.

**Vietnam**						
**Year**	**Heterogeneous**		**Homogeneous**			
	r_ω	**incidence target**	r_ω		**incidence**	
			**implemented**	**perceived**	**target**	**achieved**
1	–0.1018	178.3	–0.0701	–0.0510	178.9	181.6
2	–0.1018	167.3	–0.0724	–0.0509	170.6	173.5
3	–0.1018	156.4	–0.0753	–0.0523	162.1	165.0
4	–0.1018	145.9	–0.0789	–0.0551	153.4	156.3
5	–0.1018	135.9	–0.0833	–0.0586	144.6	147.4
6	–0.1018	126.5	–0.0888	–0.0620	135.7	138.5
7	–0.1018	117.6	–0.0963	–0.0664	126.6	129.6
8	–0.1018	109.4	–0.1077	–0.0730	117.2	120.4
9	–0.1018	101.6	–0.1278	–0.0841	107.1	110.7
10	–0.1018	**94.50**	–0.1789	–0.1115	**94.50**	99.45
**Brazil**						
**Year**	**Heterogeneous**		**Homogeneous**			
	r_ω	**incidence target**	r_ω		**incidence**	
			**implemented**	**perceived**	**target**	**achieved**
1	–0.1223	49.4	–0.0732	–0.0484	49.6	50.4
2	–0.1223	46.7	–0.0764	–0.0434	47.7	48.6
3	–0.1223	43.9	–0.0811	–0.0469	45.6	46.6
4	–0.1223	41.1	–0.0869	–0.0519	43.5	44.5
5	–0.1223	38.3	–0.0938	–0.0569	41.2	42.1
6	–0.1223	35.6	–0.1027	–0.0627	38.7	39.7
7	–0.1223	33.0	–0.1150	–0.0694	36.1	37.2
8	–0.1223	30.5	–0.1343	–0.0790	33.3	34.5
9	–0.1223	28.2	–0.1703	–0.0948	30.2	31.6
10	–0.1223	**26.0**	–0.2687	–0.1321	**26.0**	28.1
**Portugal**						
**Year**	**Heterogeneous**		**Homogeneous**			
	r_ω	**incidence target**	r_ω		**incidence**	
			**implemented**	**perceived**	**target**	**achieved**
1	–0.0909	46.7	–0.0730	–0.0608	46.7	47.1
2	–0.0909	44.2	–0.0746	–0.0540	44.5	45.0
3	–0.0909	41.6	–0.0776	–0.0578	42.2	42.8
4	–0.0909	38.9	–0.0809	–0.0627	39.9	40.3
5	–0.0909	36.3	–0.0844	–0.0670	37.4	37.9
6	–0.0909	33.7	–0.0886	–0.0716	34.9	35.3
7	–0.0909	31.2	–0.0938	–0.0765	32.4	32.8
8	–0.0909	28.8	–0.1010	–0.0827	29.9	30.2
9	–0.0909	26.6	–0.1128	–0.0915	27.3	27.7
10	–0.0909	**24.5**	–0.1401	–0.1102	**24.5**	24.9

Supplementary Table 2: Decay rates in reactivation (ω) for model trajectories with contact heterogeneity and clearance of infection upon successful treatment (depicted in Fig. 4).

Country	Parameter Values		
	**Parameter**	**Heterogeneous model**	**Homogeneous model**
Vietnam	r_ω	–0.0335	–0.0243
Brazil	r_ω	–0.0271	–0.0185
Portugal	r_ω	–0.0716	–0.0593

Supplementary Table 3: Estimated decay rates (mean and 95% credible intervals) in disease progression (ϕ) and reactivation (ω) for model trajectories with contact heterogeneity and clearance of infection upon successful treatment (depicted in Fig. 5).

Country	Parameter Values						
		Heterogeneous model			Homogeneous model		
	Parameter	*Mean*	*Lower*	*Upper*	*Mean*	*Lower*	*Upper*
Vietnam	r_{ϕ}	–0.02665	–0.02667	–0.02662	–0.01103	–0.01117	–0.01088
	r_{ω}	–0.00178	–0.00179	–0.00175	–0.02059	–0.02063	–0.02054
Brazil	r_{ϕ}	–0.00472	–0.00475	–0.00469	–0.02516	–0.02536	–0.02497
	r_{ω}	–0.01169	–0.01177	–0.01163	–0.00579	–0.00589	–0.00568
Portugal	r_{ϕ}	–0.10419	–0.11043	–0.10410	–0.19799	–0.19831	–0.19768
	r_{ω}	–0.00205	–0.00207	–0.00202	–0.01198	–0.01202	–0.01194

Supplementary Table 4: Scale-up of control efforts for meeting End TB incidence targets of 90% reduction by 2035, estimated under contact heterogeneity using the 95% credible intervals for $\boldsymbol{r_\phi}$ and $\boldsymbol{r_\omega}$ (intervals include the mean values displayed in Figs. 5, 6 and 7).

**Country**	**Scale-up of control efforts ($\boldsymbol{\kappa}$)**							
	**Model with heterogeneity in**						**Homogeneous model**	
	**contacts**		**susceptibility**		**progression**			
	**lower**	**upper**	**lower**	**upper**	**lower**	**upper**	**lower**	**upper**
Vietnam ($\omega = 0.0039$)								
total clearance ($\theta = 1$)	67.74	68.91	9.89	9.94	-	-	6.11	6.15
no clearance ($\theta = 0$)	19.94	20.08	8.24	8.27	-	-	6.72	6.74
Brazil ($\omega = 0.0013$)								
total clearance ($\theta = 1$)	8.58	8.74	9.75	10.2	-	-	19.42	20.19
no clearance ($\theta = 0$)	8.72	8.84	10.56	10.7	-	-	11.02	11.34
Portugal ($\omega = 0.0013$)								
total clearance ($\theta = 1$)	64.29	65.59	72.19	73.37	14.67	14.84	11.55	11.66
no clearance ($\theta = 0$)	68.79	72.48	55.49	56.40	10.06	10.11	8.79	8.82

Supplementary Table 5: Estimated improvement rates (mean and 95% credible intervals) in successful treatment (τ) and reactivation (ω) for model trajectories with contact heterogeneity and clearance of infection upon successful treatment (depicted in Supplementary Fig. 2).

Country	Parameter Values						
		**Heterogeneous model**			**Homogeneous model**		
	**Parameter**	***Mean***	***Lower***	***Upper***	***Mean***	***Lower***	***Upper***
Vietnam	r_τ	0.00074	0.00073	0.00076	0.03667	0.03604	0.03729
	r_ω	–0.03299	–0.03302	–0.03297	–0.01386	–0.01402	–0.01369
Brazil	r_τ	0.00539	0.00536	0.00542	0.01723	0.01700	0.01746
	r_ω	–0.01231	–0.01238	–0.01225	–0.01063	–0.01076	–0.01049
Portugal	r_τ	0.11664	0.11638	0.11690	0.30349	0.30276	0.30422
	r_ω	–0.00566	–0.00576	–0.00557	–0.00296	–0.00300	–0.00292

Supplementary Table 6: Estimated decay rates (mean and 95% credible intervals) in effective contacts (β) and reactivation (ω) for model trajectories with contact heterogeneity and clearance of infection upon successful treatment (Supplementary Fig. 3).

**Country**	**Parameter Values**						
		**Heterogeneous model**			**Homogeneous model**		
	**Parameter**	***Mean***	***Lower***	***Upper***	***Mean***	***Lower***	***Upper***
Vietnam	r_β	–0.00079	–0.00080	–0.00077	–0.05395	–0.05417	–0.05370
	r_ω	–0.03283	–0.03285	–0.03281	–0.00782	–0.00788	–0.00777
Brazil	r_β	–0.00514	–0.00517	–0.00511	–0.01967	–0.01987	–0.01945
	r_ω	–0.01197	–0.01203	–0.01189	–0.00859	–0.00863	–0.00838
Portugal	r_β	–0.12426	–0.12447	–0.12406	–0.26845	–0.26897	–0.26794
	r_ω	–0.00355	–0.00358	–0.00351	–0.00359	–0.00363	–0.00355

Supplementary Table 7: Scale-up of control efforts for meeting End TB incidence targets of 90% reduction by 2035, estimated under contact heterogeneity using the 95% credible intervals for parameter pairs (r_τ, r_ω) or (r_β, r_ω) (intervals include the mean values displayed in Supplementary Figs. 2 and 3).

**Country**	**Scale-up of control efforts (κ)**							
	**Rate parameters: r_τ and r_ω**				**Rate parameters: r_β and r_ω**			
	**Heterogeneous model**		**Homogeneous model**		**Heterogeneous model**		**Homogeneous model**	
	**lower**	**upper**	**lower**	**upper**	**lower**	**upper**	**lower**	**upper**
Vietnam ($\omega = 0.0039$)	6.496	6.549	8.510	8.721	6.447	6.516	15.67	15.90
Brazil ($\omega = 0.0013$)	7.869	7.968	9.937	10.22	8.240	8.349	12.40	12.73
Portugal ($\omega = 0.0013$)	21.30	22.10	45.92	47.54	35.08	35.84	38.09	39.22

Supplementary Table 8: Estimated decay rates (mean and 95% credible intervals) in disease progression (ϕ) and reactivation (ω) for model trajectories with susceptibility heterogeneity and clearance of infection upon successful treatment (depicted in Fig. 6).

**Country**	**Parameter Values**						
		$\boldsymbol{\theta = 1}$			$\boldsymbol{\theta = 0}$		
	**Parameter**	***Mean***	***Lower***	***Upper***	***Mean***	***Lower***	***Upper***
Vietnam	r_ϕ	–0.02402	–0.02407	–0.02397	–0.02203	–0.02208	–0.02197
	r_ω	–0.01161	–0.01163	–0.01158	–0.01419	–0.01421	–0.01416
Brazil	r_ϕ	–0.00787	–0.00791	–0.00783	–0.00867	–0.00871	–0.00863
	r_ω	–0.00959	–0.00966	–0.00954	–0.00928	–0.00933	–0.00922
Portugal	r_ϕ	–0.13661	–0.13669	–0.13652	–0.15881	–0.15891	–0.15871
	r_ω	–0.00189	–0.00190	–0.00187	–0.00251	–0.00253	–0.00249

Supplementary Table 9: Estimated decay rates (mean and 95% credible intervals) in disease progression (ϕ) and reactivation (ω) for model trajectories with progression heterogeneity and clearance of infection upon successful treatment (depicted in Fig. 7).

**Country**	**Parameter Values**						
		$\boldsymbol{\theta = 1}$			$\boldsymbol{\theta = 0}$		
	**Parameter**	***Mean***	***Lower***	***Upper***	***Mean***	***Lower***	***Upper***
Portugal	r_ϕ	–0.23138	–0.23138	–0.23062	–0.22104	–0.22144	–0.22063
	r_ω	–0.01382	–0.01385	–0.01378	–0.00951	–0.00956	–0.00947

Supplementary Table 10: Estimated decay rates (mean and 95% credible intervals) in disease progression (ϕ) and reactivation (ω) for model trajectories with contact heterogeneity and no clearance of infection upon successful treatment (depicted in Supplementary Fig. 4).

**Country**	**Parameter Values**						
		**Heterogeneous model**			**Homogeneous model**		
	**Parameter**	***Mean***	***Lower***	***Upper***	***Mean***	***Lower***	***Upper***
Vietnam	r_ϕ	–0.02394	–0.02397	–0.02391	–0.02427	–0.02440	–0.02414
	r_ω	–0.00610	–0.00612	–0.00608	–0.01889	–0.01893	–0.01887
Brazil	r_ϕ	–0.00491	–0.00493	–0.00488	–0.00620	–0.00627	–0.00613
	r_ω	–0.01057	–0.01064	–0.01051	–0.01098	–0.01119	–0.01083
Portugal	r_ϕ	–0.12514	–0.12524	–0.12505	–0.19381	–0.19409	–0.19353
	r_ω	–0.00200	–0.00202	–0.00199	–0.01554	–0.01558	–0.01552

Supplementary Table 11: Prevalence of latent TB infection in 2014 according to the heterogeneous (heterogeneity in contact rates) and homogeneous models as well as independent estimates by WHO region.

**Country**	**Prevalence of LTBI (%)**				
	**Heterogeneous[1]**			**Homogeneous[1]**	**WHO region estimate[2]**
	**contacts**	**susceptibility**	**progression**		
Vietnam ($\omega = 0.0039$)					SEA
total clearance ($\theta = 1$)	27.0	27.2	-	35.3	30.8 [28.3-34.8]
no clearance ($\theta = 0$)	**28.9**	-	-	37.9	
Brazil ($\omega = 0.0013$)					AMR
total clearance ($\theta = 1$)	**15.2**	**15.3**	-	22.7	11.0 [7.0-20.0]
no clearance ($\theta = 0$)	**16.1**	-	-	24.2	
Portugal ($\omega = 0.0013$)					EUR
total clearance ($\theta = 1$)	**16.9**	**17.1**	20.9	20.9	13.7 [9.8-19.8]
no clearance ($\theta = 0$)	**18.0**	-	-	22.2	

[1] Model estimates from trajectories in Figs. 5, 6 and 7. Values that lie within confidence intervals obtained independently for the respective regions are in bold.

[2] Independent estimates from Houben, R. M. G. J. & Dodd, P. J. The global burden of latent tuberculosis infection: A re-estimation using mathematical modelling. *PLOS Med.* **13**, e1002152 (2016).
